# Nanopore and Illumina Genome Sequencing of *Fusarium oxysporum* f. sp. *lini* Strains of Different Virulence

**DOI:** 10.3389/fgene.2021.662928

**Published:** 2021-06-17

**Authors:** Ekaterina M. Dvorianinova, Elena N. Pushkova, Roman O. Novakovskiy, Liubov V. Povkhova, Nadezhda L. Bolsheva, Ludmila P. Kudryavtseva, Tatiana A. Rozhmina, Nataliya V. Melnikova, Alexey A. Dmitriev

**Affiliations:** ^1^Engelhardt Institute of Molecular Biology, Russian Academy of Sciences, Moscow, Russia; ^2^Moscow Institute of Physics and Technology, Moscow, Russia; ^3^Federal Research Center for Bast Fiber Crops, Torzhok, Russia

**Keywords:** *Fusarium oxysporum*, flax pathogen, different virulence, genome sequencing, nanopore sequencing, *de novo* genome assembly

## Introduction

*Fusarium oxysporum* f. sp. *lini* is the one of notoriously known pathogens of flax (*Linum usitatissimum* L.), causing wilt. In the case of young seedlings, the disease may lead to complete yield losses (Kommedahl et al., 1970). At the same time, flax is an important crop used for manufacturing oil of high nutritional value, food supplements, industrial products, and fiber (Jhala and Hall, 2010; Czemplik et al., 2011; Kezimana et al., 2018; Parikh and Pierce, 2019). However, flax production is dependent on the resistance of flax varieties to phytopathogens, whereas *F. oxysporum* demonstrates considerable genetic diversity (Edel et al., 2001; Michielse and Rep, 2009).

At present, phytopathogenic strains of *F. oxysporum* are classified into numerous *formae speciales* according to their ability to colonize different hosts (Armstrong and Armstrong, 1981). Unfortunately, this system cannot provide researchers with enough information on a type of the pathogen and the severity of the infection it causes (Edel-Hermann and Lecomte, 2019). Like other representatives of the species, *forma specialis lini* is morphologically and genetically heterogeneous. Strains of the pathogen differ in such characteristics as the type of sporulation, conidia formation, pigment production, and the rate of growth on different media. Moreover, the degree of pathogenicity varies within the *forma* and depends on the infected crop variety (Kommedahl et al., 1970).

For molecular classification of *F. oxysporum*, numerous markers were used (Baayen et al., 2000; Lievens et al., 2008; Baysal et al., 2010; Sharma et al., 2014; van Dam et al., 2018; Srinivas et al., 2019; Sasseron et al., 2020), and genes associated with virulence were considered as targets for molecular discrimination of strains of the fungus (Lievens et al., 2008; van Dam et al., 2018). It was shown that s*ecreted in xylem* (*SIX*) genes are associated with pathogenicity of *F. oxysporum*, and the majority of them are distributed within the sequence of one chromosome (Rep et al., 2004; Houterman et al., 2007; Kashiwa et al., 2017; Carvalhais et al., 2019). Importantly, the combination of these genes differs between and within *formae* (Lievens et al., 2009). *Secreted in xylem* are also responsible for the resistance of cultivars to certain pathogen races due to gene-for-gene interaction between *SIX* genes of a pathogen and R genes (resistance genes) of a plant. Breaking the resistance of plant lineages to wild-type *F. oxysporum* could be accomplished by deletion of a certain *SIX* gene, which is recognized by the immune system of a plant (Houterman et al., 2008). However, in the case of *F. oxysporum* f. sp. *lini*, the connection between the degree of pathogenicity of a strain and the set of its *SIX* genes is not investigated deeply, and gaining insights into the mechanisms of pathogenicity offers an opportunity for effective disease control.

Besides, *F. oxysporum* representatives differ not only in the content of genes and their sequences but also in the number of chromosomes due to chromosomal rearrangements and the mobility of lineage-specific chromosomes (Davière et al., 2001; Ma et al., 2010; Schmidt et al., 2013; Vlaardingerbroek et al., 2016; Wang et al., 2020). Having learned the statistics of deposited *F. oxysporum* assemblies, one may conclude that genome sizes vary from about 50 to 70 Mb and differ between many *formae* (data of the NCBI Genome database, https://www.ncbi.nlm.nih.gov/genome/browse/#!/eukaryotes/707/).

Summing up, *F. oxysporum* f. sp. *lini* appears to be heterogeneous. However, there is a lack of next-generation sequencing data concerning the genome structure of the flax pathogen and the molecular basis of pathogenicity in relation to diverse virulence of the strains. In this work, we have chosen six strains of *F. oxysporum* f. sp. *lini* of low, medium, and high virulence (two strains per each degree of pathogenicity), performed genome sequencing on Oxford Nanopore Technologies (ONT) and Illumina platforms, and obtained *de novo* assemblies of the sequenced strains.

## Materials and Methods

### Fungal Material

*F. oxysporum* f. sp. *lini* samples were provided by the Institute for Flax (Torzhok, Russia). The strains were of the following pathogenicity degrees: the low (strains #456, #482), the medium (#476, #525), and the high one (#483, #39). Mycelium was grown in test tubes on potato dextrose agar medium for 3 weeks (Alpha Biosciences, USA).

### DNA Extraction and Purification

Following the previously developed protocol, pure high-molecular-weight DNA of the fungal samples was obtained (Krasnov et al., 2020). In brief, the DNA was extracted using the CTAB method followed by its isolation with Blood and Cell Culture DNA Mini Kit (Qiagen, USA). The quality and concentration of the DNA were evaluated on a NanoDrop 2000C spectrophotometer (Thermo Fisher Scientific, USA) and a Qubit 2.0 fluorometer (Life Technologies, USA). The assessment of DNA length and the control of RNA absence were performed by electrophoresis in a 0.8% agarose gel (Lonza, Switzerland).

### DNA Library Preparation and Sequencing on the Oxford Nanopore Technologies Platform

Before library preparation, DNA fragments up to 10 kb were removed from the samples with a Short Read Eliminator Kit (Circulomics, USA), and the remaining DNA was purified with AMPure XP beads (Beckman Coulter, USA) in a ratio of 1:0.7 (sample:beads). SQK-LSK109 Ligation Sequencing Kit (ONT, UK) for 1D genomic DNA sequencing was used to prepare the library. During this procedure, minor modifications were introduced to the recommended protocol that included sample barcoding with the EXP-NBD103 (Native Barcoding Expansion) kit (ONT). Namely, at the steps of DNA recovery at 20°C and ligation the time of incubation was increased to 20 and 60 min respectively. Sequencing was performed on a MinION (ONT) instrument with a FLO-MIN-106D (R9.4.1) flow cell (ONT).

### DNA Library Preparation and Sequencing on the Illumina Platform

Upon DNA shearing on a S220 ultrasonic homogenizer (Covaris, USA), the library was prepared from 1 μg of fragmented DNA with the NEBNext Ultra II DNA Library Prep Kit for Illumina (New England Biolabs, UK) according to the manufacturer's protocol with size selection of adaptor-ligated DNA of about 600–800 bp. A 2100 Bioanalyzer instrument (Agilent Technologies, USA) and a Qubit 2.0 fluorometer (Life Technologies) were used to evaluate the quality and concentration of the DNA library respectively. The DNA library was sequenced on a MiSeq instrument (Illumina, USA) with a read length of 300+300 bp.

### Preliminary Data Analysis

On genome sequencing of five strains (#456, #476, #482, #483, #525), we obtained 2.7 Gb of ONT data (374–756 Mb per strain, N50 = 32–44 kb) and 26.7 million Illumina paired-end reads (2.9–7.9 million 300+300 bp reads per strain). Since *F. oxysporum* f. sp. *lini* genome size is about 60–70 Mb (Kanapin et al., 2020; Krasnov et al., 2020), the obtained data corresponded to 10x genome coverage with ONT reads and 50x coverage with Illumina reads on average. The Nanopore and Illumina sequencing data for these five strains and isolate #39, which was sequenced by us earlier, were deposited in NCBI under the BioProject accession number PRJNA721899. For further use in performing *de novo* genome assemblies of the five sequenced strains, ONT fast5 files were basecalled with Guppy 3.6.1 (https://community.nanoporetech.com/protocols/Guppy-protocol/v/GPB_2003_v1_revU_14Dec2018) using the dna_r9.4.1_450bps_hac.cfg config file. Adapter removal and demultiplexing were carried out with Porechop 0.2.4 (https://github.com/rrwick/Porechop). Reads with an average quality below 6 (*Q* < 6) were discarded with Trimmomatic 0.39 (Bolger et al., 2014). To perform the initial genome assemblies, we chose the Canu tool (version 2.1) developed for long-read datasets (Koren et al., 2017), as it provided us with the best results when assembling the genome of isolate #39 in our previous study (Krasnov et al., 2020). The approximate size of the genomes was set as 60 Mb, and the other Canu parameters were kept default. The resulting quality of the assemblies was judged by QUAST features (version 5.0.2) (Mikheenko et al., 2018) and BUSCO completeness (version 4.1.2, hypocreales_odb10 dataset) (Seppey et al., 2019). BUSCO and QUAST statistics for the Canu genome assemblies of the five strains were the following: completeness laid in a range of 61.9–94.5%, N50 was 0.2–2.0 Mb, L50 varied between 9 and 82 (Table 1, Canu). These assemblies were not good enough for further analysis that can be explained by the low coverage of genomes with ONT reads (about 6–13x). So, we decided to perform hybrid assemblies of the genomes of the five strains with MaSuRCA (version 3.4.2, CA algorithm) (Zimin et al., 2013) using both ONT and Illumina data, as the obtained coverage with Illumina reads was high (30–80x) and MaSuRCA showed decent results for isolate #39 earlier (Krasnov et al., 2020). The N50 parameter of the hybrid assemblies of strains #456, #476, #482, #483, and #525 laid in a range of 1.0–2.5 Mb, L50 was 7–21, and the BUSCO completeness varied from 96.5 to 99.7% (Table 1, MaSuRCA). These statistics indicate fairly high contiguity and completeness of the obtained MaSuRCA assemblies. They were deposited in NCBI under the BioProject accession number PRJNA721899. Besides, we tested if ONT reads were crucial for assembly quality and assembled the genome of strain #525 from only Illumina reads using MaSuRCA, as the smallest amount of ONT data was obtained for this strain (0.37 Mb). It appeared that even 5–10x coverage of a genome with long ONT reads significantly improved assembly contiguity—N50 = 961 kb and L50 = 21 for the hybrid assembly against N50 = 92 kb and L50 = 145 for the assembly from Illumina reads only. Each of the received assemblies of the five strains contained a circular contig representing a complete mitochondrial genome which can be used in phylogenetic studies of *Fusarium* species. The obtained assemblies are a useful resource for comparative genomic studies of the flax pathogen strains.

**Table 1:** QUAST and BUSCO statistics for Canu and MaSuRCA genome assemblies of six *F. oxysporum* f. sp. *lini* strains.

**Strain number**		**456**	**482**	**476**	**525**	**483**	**39**
**Pathogenicity**		**low**		**medium**		**high**	
ONT data volume, Gb^*^		0.38	0.76	0.59	0.37	0.56	4.87
**Canu assemblies**							
QUAST	Number of contigs	338	53	191	323	226	36
statistics	Total length, Mb	51.4	51.1	64.0	49.2	63.4	69.5
	N50, kb	204	2,036	634	193	436	4,152
	L50	80	9	35	82	41	6
	GC, %	48.05	47.70	47.95	48.28	47.95	47.99
BUSCO	Complete, %	68.0	94.5	85.6	61.9	86.4	97.8
statistics	Single, %	67.4	93.9	84.8	61.3	85.6	96.8
	Duplicated, %	0.6	0.6	0.8	0.6	0.8	1.0
**MaSuRCA assemblies**							
QUAST	Number of contigs	113	43	67	123	97	74
statistics	Total length, Mb	61.7	53.4	65.9	63.1	63.8	69.3
	N50, kb	1,063	2,363	2,485	961	1,594	2,336
	L50	17	7	9	21	11	5
	GC, %	47.86	47.30	47.87	48.11	47.98	48.07
BUSCO	Complete, %	96.5	99.7	99.1	98.2	98.4	99.7
statistics	Single, %	95.0	98.0	97.5	97.2	97.2	97.9
	Duplicated, %	1.5	1.7	1.6	1.0	1.2	1.8

* *The volume of ONT data after basecalling with Guppy, adapter trimming with Porechop, and quality filtration with Trimmomatic is presented*.

The comparison of the *F. oxysporum* f. sp. *lini* strains studied in the present work was also performed based on Illumina reads mapped to reference genomes. We trimmed Illumina reads of the six strains with Trimmomatic 0.39 (trailing:28) and discarded the short ones (minlen:50); the remaining data were mapped to reference assemblies of *F. oxysporum* using Bowtie 2 (version 2.3.5.1) (Langmead et al., 2019), which also reported the overall alignment rate, and the alignments were sorted with samtools (version 1.10) (Li et al., 2009). Samtools was also used to determine the coverage of contigs by Illumina reads in each assembly, and the overall coverage was calculated as the sum of covered bases in each contig divided by the number of all bases in an assembly. The only two complete *F. oxysporum* genomes deposited in GenBank were downloaded to be used for mapping—the assembly of strain Fo5176 (GCA_013112355.1, https://www.ncbi.nlm.nih.gov/genome/707?genome_assembly_id=1472496) by Fokkens et al. (2020) and the assembly of strain Fo47 (GCA_013085055.1, https://www.ncbi.nlm.nih.gov/genome/707?genome_assembly_id=910449) by Wang et al. (2020). Besides, the genome of *F. oxysporum* f. sp. *lini* isolate #39 was reassembled with Canu, using the earlier obtained data (Krasnov et al., 2020) and according to the same strategy as described above (ONT read processing with Guppy, Porechop, Trimmomatic, assembling with Canu), and with MaSuRCA (basecalling with Guppy, assembling from ONT and Illumina reads with MaSuRCA) (Table 1). Polishing of the Canu assembly was conducted using ONT reads according to the previously optimized scheme—two iterations with the Racon polisher and one with Medaka (Dmitriev et al., 2020). The accuracy of the Canu assembly was also improved by polishing with POLCA (from MaSuRCA) using Illumina reads (Zimin and Salzberg, 2020). The MaSuRCA assembly does not need additional polishing (Zimin et al., 2017). The resulting Canu assembly consisted of 41 contigs with N50 of 4.15 Mb, had a length of 69.5 Mb, and completeness of 99.8%. As expected, the polished Canu assembly outperformed the MaSuRCA one in terms of QUAST and BUSCO statistics, so the Canu-assembled and polished genome of *F. oxysporum* f. sp. *lini* isolate #39 was used for further mapping.

Illumina reads of the six differently virulent strains of *F. oxysporum* f. sp. *lini* were separately mapped to the two complete genomes of strains Fo5176 and Fo47 and also the assembly of isolate #39 obtained anew (Table 2). The overall alignment of the mapped reads corresponding to the six strains against the genomes of isolate #39 and strains Fo5176 and Fo47 as well as the percentages of the genome covered were higher for isolate #39 than for Fo5176 and Fo47. Fo5176 infects *Arabidopsis thaliana* (Fokkens et al., 2020) and has a comparable to isolate #39 genome size (68.0 and 69.5 Mb respectively), while Fo47 is classified as an endophyte and has a significantly smaller genome size−50.4 Mb (Wang et al., 2020). The percentages of aligned reads and covered genome fractions varied between *F. oxysporum* f. sp. *lini* strains; however, we did not reveal the similarity between genomes of the strains with the same pathogenicity degree. For example, high-virulent isolate #39 had less resemblance to another high-virulent strain #483 than to strains #476 and #525 with medium virulence. At the same time, low-virulent strain #482 had the most significant difference from isolate #39. Thus, mapping our Illumina sequencing data of differently virulent strains enabled us to evaluate broad-scale differences in genomes. Besides, using the present Illumina data, one can also reveal single nucleotide polymorphisms (SNPs) or small insertions/deletions in the loci of interest and perform a comparison of the *F. oxysporum* f. sp. *lini* strains with *F. oxysporum* strains of other *formae speciales*. At the same time, the genomes of the six studied strains, even of those ones with similar virulence, differed in the amount of mapped Illumina data, and, therefore, mapping Illumina reads to a reference genome can result in the loss of significant part of information. Besides, chromosomal rearrangements may be implicated in gene regulation, and, for evaluation of the role of such variations in fungal pathogenicity, *de novo* genome assemblies obtained from long reads are indispensable (Demené et al., 2021). Thus, both Illumina and ONT data obtained by us for *F. oxysporum* f. sp. *lini* strains of different virulence are essential for a comprehensive comparative analysis of the genomes and identification of genetic differences associated with pathogenicity.

**Table 2:** Percentages of aligned Illumina reads against the assembly of *F. oxysporum* f. sp. *lini* isolate #39 and the complete genomes of *F. oxysporum* strains Fo47 and Fo5176 and percentages of the genomes covered by Illumina reads for six *F. oxysporum* f. sp. *lini* strains.

**Strain number**		**456**	**482**	**476**	**525**	**483**	**39**
**Pathogenicity**		**low**		**medium**		**high**	
Illumina data volume, Gb		4.76	3.22	3.32	3.00	1.72	1.78
**Genome to be aligned against**	**Genome** **size, Mb**	**Percentage of aligned reads, %**					
*F. oxysporum* f. sp. *lini* #39	69.5	82.85	78.48	83.45	93.17	83.99	95.03
*F. oxysporum* Fo5176 (GCA_013112355.1)	68.0	74.30	76.72	74.44	78.07	75.23	77.09
*F. oxysporum* Fo47 (GCA_013085055.1)	50.4	63.75	75.11	63.79	63.11	65.35	62.87
**Genome to be covered**	**Genome** **size, Mb**	**Percentage of a genome covered, %**					
*F. oxysporum* f. sp. *lini* #39	69.5	89.29	71.40	95.97	95.51	88.34	99.34
*F. oxysporum* Fo5176 (GCA_013112355.1)	68.0	82.74	71.02	85.75	83.26	80.94	95.84
*F. oxysporum* Fo47 (GCA_013085055.1)	50.4	88.23	85.61	89.90	84.69	87.82	83.54

Another example of the use of the obtained dataset is the identification of genes of interest and their further analysis. For instance, we performed the search for genes involved in a process of plant colonization. The blastn analysis showed that the obtained assemblies of all the strains but low-virulent #482 contained regions with high homology (*e*-value < 10^−10^, identity of 99% on average) to partial coding sequences of virulence genes found in *forma specialis lini*: *SIX1* (NCBI: KM893920.1), *SIX7* (KM893928.1), *SIX10* (KP964982.1), *SIX12* (KP964992.1), *SIX13* (KP964998.1) (Laurence et al., 2015; Taylor et al., 2016). The identified *SIX* sequences were found arranged in clusters. For example, in the genome of isolate #39, partial coding sequences of *SIX1, SIX7, SIX10, SIX13* mapped completely against the locus of 400 kb of tig00000022, and *SIX7* mapped twice against this locus. Besides, homologs of *SIX7, SIX12* sequences were present in tig00000001, and those of *SIX7, SIX10, SIX12, SIX13* were found in a 100 kb locus of tig00000029. Similar clustering of *SIX* sequences was observed in the assemblies of strains #456, #476, #483, and #525. Thus, the obtained data are valuable for identifying genes of a particular family, including those responsible for pathogenicity and playing an important role in the interaction between *F. oxysporum* and plants.

One more illustration of a way to use our dataset is the study of the role of DNA modifications in the regulation of *F. oxysporum* genome. DNA methylation is implicated in gene expression regulation, repression of transposable elements, and chromatin remodeling. To date, it is known that cytosines in fungi can be methylated within the context of CpG sites, CN (N for any nucleotide) pairs, and long clusters of cytosines; the type of the most methylated motif varies between fungal species, while the overall level of methylation in fungal genomes is relatively low (He et al., 2020). Several approaches of the ONT data use for the evaluation of methylation level throughout the genome of an object were developed (Xu and Seki, 2020; Tourancheau et al., 2021). We assessed DNA methylation levels in the genome of isolate #39, for which high coverage with ONT reads was obtained (about 70x), using the nanopolish tool (https://nanopolish.readthedocs.io/en/latest/quickstart_call_methylation.html), which is trained for methylation evaluation within the CpG context. The basecalled ONT reads were mapped against the assembly of isolate #39 with minimap2, alignments were derived using samtools, and methylation levels of CpG sites were estimated with nanopolish. Methylation across the whole genome was low (Supplementary Data 1) that is in concordance with other studies on DNA methylation in fungi (Bewick et al., 2019). Nevertheless, in the assembly of *F. oxysporum* f. sp. *lini* isolate #39, more than 500 CpG sites (with coverage >20 ONT reads) had methylation levels ≥0.5. Then, due to poor annotation of *F. oxysporum* genomes, we performed a blast-search for the regions containing these CpG sites in fungi taxa. Most of the methylated CpG sites were not assigned to specific functional elements of the genome; however, there were also CpG sites that were located in promoter regions, transposons, and gene bodies. Therefore, our dataset is useful for the evaluation of the role of DNA methylation in genome regulation of *F. oxysporum* f. sp. *lini*.

Finally, the obtained genome assemblies of *F. oxysporum* f. sp. *lini* strains can be used in gene expression studies, for example, as a reference for transcriptome assembly with further expression analysis or in selection of conservative regions suitable for primer design for the assessment of gene expression by quantitative PCR in different *F. oxysporum* strains or even different *Fusarium* species.

## Conclusions

This work mainly focused on genome sequencing of strains of the flax pathogen *F. oxysporum* f. sp. *lini*, possessing diverse pathogenicity degrees, on two platforms—ONT and Illumina. The collected data allowed us to assemble the genomes of five strains and reassemble the genome of isolate #39 (the used data were obtained in our previous work), which lay the basis for further investigation of *F. oxysporum* virulence mechanisms and contribute to understanding the general structure of the pathogen population. Due to *F. oxysporum* f. sp. *lini* includes a vast number of genotypes, it is of high significance to study the origins of pathogenicity at molecular level. Our dataset can be of great use for researchers working on breeding resistant flax varieties and developing methods to prevent the disease and economic losses.

## Data Availability Statement

The obtained data can be found in NCBI under the BioProject accession number PRJNA721899.

## Author Contributions

TR, NM, and AD conceived and designed the work. ED, EP, RN, LP, NB, and LK performed the experiments. ED, RN, TR, NM, and AD analyzed the data. ED, NM, and AD wrote the manuscript. All authors contributed to the article and approved the submitted version.

## Conflict of Interest

The authors declare that the research was conducted in the absence of any commercial or financial relationships that could be construed as a potential conflict of interest.

## Supplementary Material

Supplementary Data 1 Methylation levels of CpG sites in the genome assembly of *F. oxysporum* f. sp. *lini* isolate #39. Data derived from Nanopore sequencing data using nanopolish. Sites with at least 20x coverage are presented.
